# Recomendaciones para el tratamiento de la psoriasis en Pediatría

**DOI:** 10.31053/1853.0605.v80.n4.42874

**Published:** 2023-12-26

**Authors:** Paula Carolina Luna, María Eugenia Abad, Margarita Larralde, Paula Boggio, Bruno Ferrari, María Fernanda Maccario, Carla Castro, Silvia Moreno, Débora Kaplan, Cristina Echeverría

**Affiliations:** 1 Hospital Alemán. Servicio de Dermatología Buenos Aires Argentina; 2 Hospital Alemán. Servicio de Dermatología. Servicio de Dermatología Pediátrica Hospital Ramos Mejía Buenos Aires Argentina; 3 Hospital Italiano de Buenos Aires. Departamento de Pediatría. Sección Dermatología Infantil Buenos Aires Argentina; 4 Hospital Ramos Mejía. Servicio de Dermatología Buenos Aires Argentina; 5 Hospital de Niños V. J. Vilela. Servicio de Dermatología Pediátrica Rosario Argentina; 6 Universidad Austral. Hospital Universitario Austral. Servicio de Dermatología Pilar Argentina; 7 Hospital Pediátrico Doctor Humberto Notti Mendoza Argentina; 8 Instituto de Rehabilitación Psicofísica Buenos Aires Argentina

**Keywords:** psoriasis, guía de práctica clínica, Argentina, pediatría, sistema GRADE, psoriasis, clinical practice guideline, pediatrics, GRADE approach, psoríase, diretriz de prática clínica, Argentina, pediatria, sistema GRADE

## Abstract

**Introducción:**

un tercio de los pacientes con psoriasis comienzan con sus síntomas en la niñez y la adolescencia, con fuerte impacto emocional y psicosocial.

**Objetivo:**

elaborar una guía de tratamiento sistémico de la psoriasis en pacientes pediátricos mediante recomendaciones fundamentadas en la mejor evidencia disponible.

**Materiales y métodos:**

Fuentes: artículos indexados en PubMed, Epistemonikos, Google Académico, Cochrane Library y Scielo, publicados entre enero de 2010 y mayo de 2022, en inglés, castellano y portugués. Selección de estudios: se consideraron guías de práctica clínica basadas en la evidencia, revisiones sistemáticas, metanálisis, estudios controlados y aleatorizados, estudios observacionales (casos y controles, estudios de cohortes, registros de la vida real) y evaluaciones de medicamentos biosimilares en pacientes de hasta 17 años de edad inclusive. Se utilizaron las palabras clave "psoriasis" y "tratamiento" en los tres idiomas. Extracción de datos: la bibliografía fue evaluada mediante las recomendaciones del sistema *Grading of Recommendations Assessment, Development and Evaluation* (GRADE). Síntesis de datos: elaboración de tablas de evidencia que fueron analizadas por el comité de expertos. Las preguntas para el desarrollo de recomendaciones se fundamentaron en el sistema PICO (población, intervención, comparación, *outcome* [desenlace]).

**Resultados:**

se elaboraron un total de 8 recomendaciones y 7 puntos de buena práctica. La dirección y fuerza de las recomendaciones se expresaron de acuerdo con lo sugerido por el sistema GRADE.

**Conclusiones:**

la decisión final de una terapia específica se fundamentará en la mejor opinión del médico tratante, las características individuales, y los valores y preferencias de los pacientes y sus cuidadores.

## Abstract (en)

**Introduction:**

in one third of patients with psoriasis, symptoms start during childhood and adolescence, with a strong emotional and psychosocial impact.

**Objective:**

to develop a guideline for the systemic treatment of psoriasis in pediatric patients by means of recommendations based on the best available evidence.

**Materials and methods:**

Sources: articles indexed in PubMed, Epistemonikos, Google Scholar, Cochrane Library and Scielo, published between January 2010 and May 2022, in English, Spanish and Portuguese. Study selection: evidence-based clinical practice guidelines, systematic reviews, meta-analyses, randomized controlled studies, observational studies (case-control, cohort studies, real-life registries) and evaluations of biosimilar drugs in patients up to and including 17 years of age were considered. The keywords "psoriasis" and "treatment" were used in all three languages. Data extraction: the literature was evaluated using Grading of Recommendations Assessment, Development and Evaluation (GRADE) recommendations. Data synthesis: evidence tables were developed and analyzed by the expert committee. The questions for the development of recommendations were based on the PICO system (population, intervention, comparison, outcome).

**Results:**

A total of 8 recommendations and 7 points of good practice were developed. The direction and strength of the recommendations were expressed according to the GRADE system.

**Conclusions:**

the final decision on a specific therapy should be based on the best opinion of the treating physician, the individual characteristics, and the values and preferences of the patients and their caregivers.

## Resumo

**Introdução:**

um terço dos pacientes com psoríase começa a apresentar sintomas na infância e na adolescência, com forte impacto emocional e psicossocial.

**Objetivo:**

elaborar uma diretriz para o tratamento sistêmico da psoríase em pacientes pediátricos por meio de recomendações baseadas nas melhores evidências disponíveis.

**Materiais e métodos:**

Fontes: artigos indexados no PubMed, Epistemonikos, Google Scholar, Cochrane Library e Scielo, publicados entre janeiro de 2010 e maio de 2022, em inglês, espanhol e português. Seleção de estudos: foram consideradas diretrizes de prática clínica baseadas em evidências, revisões sistemáticas, metanálises, estudos controlados randomizados, estudos observacionais (caso-controle, estudos de coorte, registros da vida real) e avaliações de medicamentos biossimilares em pacientes com até 17 anos de idade, inclusive. As palavras-chave "psoríase" e "tratamento" foram usadas nos três idiomas. Extração de dados: a literatura foi avaliada usando as recomendações do *Grading of Recommendations Assessment, Development and Evaluation* (GRADE). Síntese de dados: as tabelas de evidências foram desenvolvidas e analisadas pelo comitê de especialistas. As perguntas para o desenvolvimento de recomendações foram baseadas no sistema PICO (população, intervenção, comparação, *outcome* [resultado]).

**Resultados:**

foram desenvolvidas 8 recomendações e 7 pontos de boas práticas. A direção e a força das recomendações foram expressas conforme sugerido pelo sistema GRADE.

**Conclusões:**

a decisão final sobre uma terapia específica será baseada no melhor julgamento do médico responsável pelo tratamento, nas características individuais e nos valores e preferências dos pacientes e de seus cuidadores.

**CONCEPTOS CLAVE**

**Que se sabe sobre el tema**

La psoriasis es una enfermedad inflamatoria crónica que afecta primariamente a la piel, pero puede estar acompañada de otras enfermedades sistémicas y causar una importante morbilidad psicosocial. Hasta el 30 % de los pacientes presentan sus primeros síntomas durante la infancia y la adolescencia, con un impacto especialmente acentuado en el bienestar emocional y psicosocial.

**Que aporta este trabajo**

Los avances en el conocimiento de la fisiopatogenia de la enfermedad han permitido que los tratamientos resulten no solo cada vez más efectivos y seguros, sino que también se investigaran específicamente en los pacientes pediátricos. Se presentan una serie de recomendaciones terapéuticas tras la evaluación de la evidencia mediante metodología GRADE.

**Divulgación**

La psoriasis es una enfermedad inflamatoria crónica que afecta primariamente a la piel y se acompaña de gran repercusión psicosocial. Hasta un tercio de los pacientes comienzan con sus síntomas en la niñez y la adolescencia, con fuerte impacto en el bienestar emocional y psicosocial. En este trabajo, efectuado bajo una metodología especialmente diseñada para evaluar y calificar a la información disponible, se presentan una serie de recomendaciones para el tratamiento de los niños y adolescentes con psoriasis.

## Introducción

La psoriasis es una enfermedad inflamatoria crónica que afecta a la piel y, en algunos casos, a las articulaciones. En los últimos años se ha reconocido que, además, puede estar acompañada de otras enfermedades sistémicas (como el síndrome metabólico, entre otras) y causar una importante morbilidad psicosocial ^1^ . Hasta el 30 % de los pacientes con psoriasis presentan sus primeros síntomas durante la infancia y la adolescencia. El impacto de la enfermedad en el bienestar emocional y psicosocial de este grupo de pacientes es especialmente acentuado, dado que las manifestaciones de la psoriasis pueden afectar significativamente la autoestima y este efecto suele persistir en la edad adulta ^2^ .

La prevalencia de la psoriasis en pediatría varía según el país y la población, como probable consecuencia de que, en esta enfermedad compleja, participan factores ambientales en sujetos genéticamente predispuestos ^3^ .

Los avances en el conocimiento de la fisiopatogenia de la enfermedad han permitido que los tratamientos resulten no solo cada vez más efectivos y seguros, sino que también se investigaran específicamente en los pacientes pediátricos. Como consecuencia, se ha avanzado enormemente en los tratamientos basados en la evidencia en este grupo etario. Las diferencias culturales y eventualmente de acceso a las distintas terapias vuelven relevante la disponibilidad de guías nacionales que se adapten a las posibilidades locales.

## Materiales y Métodos

Con la iniciativa de dos sociedades científicas abocadas al tema (la Sociedad de Dermatología Pediátrica para Latinoamérica [SDPL] y la Sociedad Argentina de Psoriasis [SOARPSO]) se conformó un panel de expertos nacionales en el tratamiento de los pacientes pediátricos con psoriasis, quienes efectuaron reuniones en formato virtual, con el objetivo de elaborar una guía de tratamiento sistémico de esta afección por medio de una serie de recomendaciones fundamentadas en la mejor evidencia disponible. Se llevó a cabo una búsqueda bibliográfica en las bases de datos PubMed, Epistemonikos, Google Académico, Cochrane Library y Scielo, con fechas de publicación digital (ePub) comprendidas entre 1 de enero del 2010 y el 30 de mayo del 2022, en idioma inglés, castellano y portugués. Se seleccionaron aquellos estudios que incluyeron pacientes de hasta 17 años de edad inclusive, y se utilizaron las palabras clave "psoriasis" y "tratamiento" en los tres idiomas. Se consideraron guías de práctica clínica basadas en la evidencia, revisiones sistemáticas cualitativas, metanálisis, estudios controlados y aleatorizados, estudios observacionales (casos y controles, estudios de cohortes, registros de la vida real) y evaluaciones de medicamentos biosimilares.

La bibliografía reunida fue evaluada mediante las recomendaciones actuales del sistema *Grading of Recommendations Assessment, Development and Evaluation* (GRADE) ^4^ , con la elaboración de tablas de evidencia que fueron analizadas por el comité de expertos. En una primera etapa se consideran de alta calidad a las síntesis de evidencia (metanálisis, revisiones sistemáticas, guías de práctica clínica basadas en la evidencia) y los estudios clínicos aleatorizados; por otra parte, se definieron como de baja calidad de evidencia a los estudios observacionales (casos y controles, cohortes, registros del mundo real). En una segunda etapa, se refinó la calificación de la evidencia mediante la valoración de los parámetros que reducen (limitaciones en el diseño, riesgo de sesgos, inconsistencia de los resultados, incertidumbre de que la evidencia sea directa, imprecisiones, sesgos de notificación) o elevan (fortaleza de la asociación, existencia de gradiente dosis-respuesta, evidencia de que todos los factores de confusión o sesgos podrían haber reducido el efecto informado) la calidad de la evidencia.

Las preguntas para el desarrollo de las recomendaciones se fundamentaron en el sistema PICO (población, intervención, comparación, *outcome* [desenlace]) propuesto en la metodología GRADE (Tabla 1). Como resultado del análisis de la evidencia y del debate entre los autores, se elaboraron un total de 8 recomendaciones y 7 puntos de buena práctica. La dirección y fuerza de las recomendaciones se expresaron de acuerdo con lo sugerido por el sistema GRADE ^5^ (Tabla 2).

Los contenidos de estas recomendaciones están dirigidos a todos los profesionales de la salud involucrados en el tratamiento de los pacientes pediátricos con psoriasis.

**Tabla N° 1:** Preguntas PICO

1. En los pacientes de hasta 17 años con psoriasis en placa, ¿cuáles son los parámetros de eficacia terapéutica a considerar al inicio y durante el seguimiento clínico (BSA, CDLQI, otros)?
2. En los pacientes de hasta 17 años con psoriasis en placa leve, ¿cuál es el tratamiento tópico de primera elección (corticoides, inhibidores de la calcineurina, análogos de la vitamina D, antralinas, coaltar), considerando eficacia y seguridad?
3. En los pacientes de hasta 17 años con psoriasis en placa moderada a severa, ¿cuál es el tratamiento tópico de primera elección (corticoides, inhibidores de la calcineurina, análogos de la vitamina D, antralinas, coaltar), considerando eficacia y seguridad?
4. En los pacientes de hasta 17 años con psoriasis en placa leve, ¿cuál es el lugar de la fototerapia en el tratamiento, considerando eficacia y seguridad?
5. En los pacientes de hasta 17 años con psoriasis en placa moderada a severa, ¿cuál es el lugar de la fototerapia en el tratamiento, considerando eficacia y seguridad?
6. En los pacientes de hasta 17 años con psoriasis en placa moderada a severa, ¿cuál es el lugar de los corticoides sistémicos en el tratamiento, considerando eficacia y seguridad?
7. En los pacientes de hasta 17 años con psoriasis en placa moderada a severa, ¿cuál es la indicación de tratamiento médico sistémico no biológico (metotrexato, ciclosporina A, retinoides, fumaratos)?
8. En los pacientes de hasta 17 años con psoriasis en placa moderada a severa, ¿cuál es el tratamiento médico sistémico no biológico de primera elección (metotrexato, ciclosporina A, retinoides, fumaratos), considerando eficacia y seguridad?
9. En los pacientes de hasta 17 años con psoriasis en placa moderada a severa, ¿cuál es la indicación de tratamiento médico sistémico biológico (adalimumab, etanercept, infliximab, ixekizumab, secukinumab, ustekinumab)?
10. En los pacientes de hasta 17 años con psoriasis en placa moderada a severa, ¿cuál es el tratamiento médico sistémico biológico de primera elección (adalimumab, etanercept, infliximab, ixekizumab, secukinumab, ustekinumab), considerando eficacia y seguridad?
11. En los pacientes de hasta 17 años con psoriasis en placa moderada a severa, ¿cuál es la indicación de tratamiento sistémico con moléculas pequeñas (apremilast), considerando eficacia y seguridad?
12. En los pacientes de hasta 17 años con psoriasis en placa leve, ¿cuál es la indicación de tratamiento con probióticos, prebióticos, acciones sobre el microbioma o su combinación, considerando eficacia y seguridad?
13. En los pacientes de hasta 17 años con psoriasis en placa moderada a severa, ¿cuál es la indicación de tratamiento con probióticos, prebióticos, acciones sobre el microbioma o su combinación?

**Tabla N° 2:** Dirección y fuerza de las recomendaciones

Recomendación fuerte ("se recomienda..."): se fundamentan en evidencia consistente acerca de beneficios netos de una intervención por sobre sus potenciales consecuencias indeseables. La mayor parte de los pacientes informados elegirían la opción recomendada.
Recomendación débil ("se sugiere..."): la evidencia de beneficios netos de la intervención es de menor calidad o la elección de los pacientes variará según sus valores y preferencias. Las consecuencias deseables de la intervención probablemente sobrepasan a los posibles efectos no deseados.
Punto de buena práctica: práctica recomendada y fundamentada en la opinión del panel de expertos.

## Resultados

**Recomendación 1** : en los pacientes de hasta 17 años con psoriasis en placa, el panel de expertos recomienda alcanzar un PGA 0-1, un PASI absoluto < 3, un PASI90 y/o un CDLQI 0-1 como objetivos de tratamiento (fuerza de la recomendación: fuerte, a favor).

Punto de buena práctica: el tiempo para alcanzar estos objetivos puede variar según el tratamiento elegido.

De acuerdo con la experiencia en adultos, el cálculo de un puntaje *Psoriasis Area and Severity Index* (PASI) absoluto ≤ 3 resulta útil, dado que es independiente de las variaciones en la severidad basal de la enfermedad y se aproxima mejor al concepto de un valor de *Physician Global Assessment* (PGA) 0–1 ^6^ . En relación a la respuesta PASI90, los expertos proponen su utilización como meta terapéutica en función de la elevada eficacia de las actuales terapias biológicas.

La herramienta *Children Dermatology Life Quality Index* (CDLQI), adaptada a los pacientes de 4 a 16 años, permite demostrar que las puntuaciones de calidad de vida se correlacionan con la gravedad de la enfermedad; además, el puntaje mejora con el éxito del tratamiento ^7^ .

**Recomendación 2** : en los pacientes de hasta 17 años con psoriasis en placa leve, se sugieren como tratamientos tópicos, en orden preferencial: la asociación de corticoides tópicos y calcipotriol; la monoterapia con corticoides; la monoterapia con calcipotriol. Los inhibidores de la calcineurina se sugieren como fármacos de elección para las lesiones en el rostro, los pliegues y los genitales (fuerza de la recomendación: débil, a favor).

Punto de buena práctica: los corticoides sugeridos de primera elección incluyen la mometasona y la betametasona en la primera infancia. Pueden considerarse otros de mayor potencia, como la metilprednisolona y la triamcinolona, en los adolescentes.

Punto de buena práctica: se sugiere consultar las recomendaciones del fabricante ( *label* ) para definir la extensión de la superficie a aplicar y el tiempo de duración del tratamiento para cada producto.

Los análogos de la vitamina D, como el calcipotriol, se utilizan en combinación con los corticoides tópicos. Una ventaja importante de estos análogos es su utilidad como de ahorradores de corticoides ^8^ . De acuerdo con una revisión sistemática con datos de 9 estudios controlados y aleatorizados (n = 4483), la asociación de análogos de la vitamina D con corticoides tópicos se asocia con resultados significativamente mejores que la monoterapia ^9^ . Este tratamiento se considera seguro, eficaz y relativamente bien tolerado en los niños, si bien, dado que pueden causar irritación, se evitan en el rostro, los genitales y los pliegues; áreas en las cuales los inhibidores de la calcineurina se sugieren como fármacos de elección ^8^ . Para los análogos de la vitamina D existe además el riesgo potencial de hipercalcemia e hipovitaminosis D por absorción sistémica ^8^ . El panel no recomienda el uso de estos fármacos en los niños menores de un año.

En cuanto a los corticoides tópicos, la mayor parte de la evidencia disponible en pacientes con psoriasis se origina a partir de estudios efectuados en población adulta. En estos casos, tanto los corticoides de alta potencia (como propionato de fluticasona) ^10^ como aquellos de baja o moderada potencia (acetónido de fluocinolona 0,01 %, valerato de betametasona) ^11, 12^ son superiores al placebo en términos del puntaje PGA y de la evaluación global. En una revisión sistemática con datos de cinco ensayos clínicos (835 pacientes) en los que se utilizó triamcinolona (un estudio), dipropionato de betametasona (3 estudios) o valerato de betametasona (un estudio), el uso de estos corticoides tópicos redujo significativamente el PASI absoluto en una media de –51,58 % (rango: –45 % a –60,5 %) con un tratamiento de 4 a 8 semanas ^13^ . Se propone que los corticoides de potencia moderada y alta potencia se reserven para los niños entre 2 y 12 años y mayores de 12 años, respectivamente ^14^ .

Los productos con coaltar pueden emplearse en la psoriasis del cuero cabelludo ^8^ , a excepción de los niños menores de un año.

**Recomendación 3** : en los pacientes de hasta 17 años con psoriasis en placa leve, la fototerapia podría considerarse como opción terapéutica en asociación con otros tratamientos (fuerza de la recomendación: débil, a favor).

**Recomendación 4** : en los pacientes de hasta 17 años con psoriasis en placa moderada a severa (por su extensión o según su localización), se sugiere el uso de fototerapia UVB (fuerza de la recomendación: débil, a favor).

Si bien la evidencia disponible en los primeros estudios clínicos para adultos ^15^ y niños ^16^ puede considerarse de baja calidad, en función del diseño metodológico, la fototerapia disminuye la inflamación cutánea, sin tener repercusión sobre la inflamación sistémica ^17^ . Este tratamiento representa una opción en los pacientes pediátricos con psoriasis moderada a severa, con un perfil de seguridad comparable al descrito en los pacientes adultos ^8^ , con eventos adversos como eritema, prurito, lesiones ampollares e hipopigmentación ^18^ .

**Recomendación 5** : en los pacientes de hasta 17 años con psoriasis en placa, el panel de expertos no recomienda el uso de corticoides sistémicos (fuerza de la recomendación: fuerte, en contra).

Si bien los corticoides sistémicos se utilizan como agentes antiinflamatorios para diversas afecciones dermatológicas, sus potenciales eventos adversos cutáneos (atrofia, estrías, telangiectasias) y especialmente sistémicos (supresión del eje adrenal, diabetes, osteoporosis, efectos cardiovasculares y gastrointestinales) son bien conocidos ^19^ . En el caso de la psoriasis, se agrega el riesgo de rebote y exacerbación de la enfermedad, así como el desarrollo de una psoriasis pustulosa generalizada tras la reducción o retiro de los corticoides sistémicos ^20^ . En este contexto de una valoración negativa de la relación entre los riesgos y los beneficios, el panel de expertos no recomienda su uso en los pacientes pediátricos con psoriasis.

**Recomendación 6** : en los pacientes de hasta 17 años con psoriasis en placa severa, el panel de expertos sugiere el uso de estos tratamientos sistémicos no biológicos en este orden preferencial: metotrexato, retinoides y ciclosporina A, por vía oral (fuerza de la recomendación: débil, a favor).

Punto de buena práctica: se sugiere reservar la ciclosporina A para los pacientes con eritrodermia, formas hiperagudas o sin respuesta a otras estrategias, durante el menor tiempo posible (hasta 12 meses).

El metotrexato es un fármaco accesible y de bajo costo, con amplia experiencia de uso en Dermatología y con datos de eficacia y seguridad a largo plazo ^8^ , sobre todo en pacientes adultos. En los análisis combinados de los datos disponibles de ensayos controlados y aleatorizados, este tratamiento se asocia con una alta probabilidad de alcanzar un PASI90 y un puntaje PGA 0–1 desde la fase de inducción ^21^ . Se ha demostrado además, en pacientes pediátricos, una mejoría de la calidad de vida estimada mediante la escala CDLQI en registros de la vida real ^22^ .

El metotrexato se indica semanalmente y puede administrarse por vía oral o subcutánea. La elevación de las transaminasas hepáticas es común y habitualmente transitoria dentro de los 3 a 4 días posteriores a la administración, por lo que los potenciales controles de laboratorio deben realizarse al menos entre 4 y 6 días después de la dosis. Los efectos adversos más graves son infrecuentes en los niños y pueden incluir mielosupresión, hepatotoxicidad y toxicidad pulmonar ^8^ . Se sugiere aconsejar a los adolescentes que reciben metotrexato acerca del riesgo de consumo de alcohol; asimismo, este fármaco está contraindicado en el embarazo ^8^ . Se recomienda el uso de ácido fólico por vía oral para minimizar el riesgo de eventos adversos.

Los retinoides representan una opción de tratamiento no inmunosupresor para los pacientes pediátricos con psoriasis, lo que puede ser relevante en situaciones específicas. Se incluyen en este grupo de fármacos la acitretina y la isotretinoína, siendo la primera el agente preferido para la psoriasis ^8^ . En dos registros clínicos con pacientes de un mes a 16 años de edad, los retinoides se asociaron con aclaramiento cutáneo en casi el 93 % de los participantes ^23, 24^ . La teratogenicidad es una preocupación importante en las adolescentes, ya que la acitretina puede permanecer almacenada en el organismo hasta 3 años, dado que se esterifica de forma inversa a etretinato. Se advierte además que el uso a largo plazo de retinoides sistémicos en niños pequeños podría asociarse con cambios óseos ^8^ .

**Recomendación 7** : en los pacientes de hasta 17 años con psoriasis en placa moderada a severa con falla a una terapia sistémica no biológica o en aquellos con contraindicación para las mismas, el panel de expertos recomienda el uso de una terapia sistémica biológica (en orden alfabético: adalimumab, etanercept, ixekizumab, secukinumab o ustekinumab, al momento de la redacción de estas recomendaciones; fuerza de la recomendación: fuerte, a favor).

Punto de buena práctica: la elección del fármaco (original o biosimilar) se fundamentará en la disponibilidad, la experiencia, la presencia de comorbilidades (incluida artritis), la frecuencia de aplicación, la edad del paciente y la aprobación vigente en el país por la entidad regulatoria, entre otras variables.

Punto de buena práctica: a estos fármacos recomendados podrán agregarse en futuras revisiones de esta guía otros agentes biológicos o moléculas pequeñas actualmente en evaluación en estudios en curso, de acuerdo con la evidencia científica y la aprobación por la entidad regulatoria.

Los medicamentos biológicos son inmunomoduladores que regulan la inflamación a través de vías específicas vinculadas con la señalización celular y el desarrollo, reclutamiento y apoptosis de las células inmunitarias. Se dispone de datos crecientes de eficacia y seguridad de estos agentes en los pacientes pediátricos, por lo cual se reconoce una mayor consideración para su uso en comparación con otros tratamientos sistémicos de primera línea ^8^ . Los agentes biológicos pueden emplearse en conjunto con corticoides tópicos.

En un metaanálisis de seis estudios con datos de 768 pacientes pediátricos, el uso de agentes biológicos se asoció con una mejoría significativa de las metas terapéuticas descritas en la recomendación 1. Para el criterio PASI90, la terapia con adalimumab, etanercept, ixekizumab, secukinumab o ustekinumab se asoció con un *odds ratio* (OR) de 14,62 (rango 3,81–56,09) en comparación ya sea con placebo o con un tratamiento no biológico. Para las metas PGA 0/1 y CDLQI 0/1, los respectivos OR fueron 12,65 (rango: 4,85–32,96) y 5,26 (3,58–7,72) ^25^ . El riesgo de eventos adversos, incluidas las infecciones y las reacciones adversas graves, fue similar para estos agentes y sus comparadores en un subanálisis que incluyó adalimumab, ixekizumab y ustekinumab ^25^ . Las reacciones adversas más frecuentes corresponden a aquellas del sitio de aplicación ^8^ .

**Recomendación 8** : para el enfoque de los pacientes de hasta 17 años con psoriasis en placa, el panel de expertos no se expresa a favor o en contra del uso de prebióticos, probióticos u otras acciones sobre el microbioma en función de la evidencia actual (sin recomendación actual).

Punto de buena práctica: esta recomendación podrá reevaluarse en futuras revisiones de esta guía de acuerdo con la creciente evidencia científica y la aprobación por la entidad regulatoria.

Se ha informado en estudios científicos recientes la existencia de disbiosis cutánea en los pacientes con psoriasis, que incluye una reducción de la riqueza y la diversidad de la microbiota en las lesiones, en comparación con las muestras obtenidas de otras áreas no afectadas o de sujetos con piel sana ^26^ . Dada la posible participación de las alteraciones de la microbiota cutánea e intestinal en la patogenia de la enfermedad, se ha sugerido un posible efecto positivo de los probióticos en estos pacientes. No obstante, al momento de la redacción de este documento, no se ha reunido la evidencia suficiente de adecuada calidad para elaborar una recomendación a favor o en contra de esta estrategia. Esta conclusión podrá modificarse en acuerdo con la disponibilidad de nuevos datos científicos.

## Discusión y conclusión

Los avances en el conocimiento de la fisiopatogenia de la psoriasis han permitido que los tratamientos basados en la evidencia resulten no solo cada vez más efectivos y seguros, sino que también se investigaran específicamente en los pacientes pediátricos. La accesibilidad a las nuevas herramientas terapéuticas en este contexto constituye un motivo para la elaboración de guías locales destinadas al complejo abordaje de la psoriasis en esta población. Por medio del abordaje GRADE ^4, 5^ , un grupo de especialistas en el tratamiento de este grupo de pacientes propone la presente serie de recomendaciones, con el objetivo de orientar a todos los profesionales de la salud involucrados en su atención, sin perder de vista que la decisión final de una terapia específica se fundamentará en la mejor opinión del médico tratante, las características individuales, y los valores y preferencias de los pacientes y sus cuidadores.
